# Rhein ameliorates inflammation, gut dysbiosis, and renal injury in obesity-related glomerulopathy mice

**DOI:** 10.3389/fphar.2025.1654062

**Published:** 2025-12-04

**Authors:** Minmin Xu, Yue Yu, Lijie Li, Kang Zhao, Jingjing Lai, Lifang Wei, Li Ge

**Affiliations:** 1 Department of Nephrology, The Third Affiliated Hospital of Fujian University of Traditional Chinese Medicine, Fuzhou, China; 2 Fujian University of Traditional Chinese Medicine, Fuzhou, Fujian, China

**Keywords:** rhein, obesity-related glomerulopathy, gut microbiota, inflammation, lipid metabolism

## Abstract

**Objective:**

Obesity-related glomerulopathy (ORG) lacks targeted therapies. Rhein, a bioactive anthraquinone from Rhei Radix et Rhizoma, was evaluated for its effects on inflammation, renal function, and gut microbiota in high-fat diet-induced ORG mice.

**Methods:**

C57BL/6J mice were fed a 60% fat diet for 12 weeks to establish ORG, followed by 300 mg/kg/day rhein free intake for 12 weeks. Serum cytokines (IL-6, TNF-α), renal histopathology, and 16S rRNA microbiome sequencing were analyzed.

**Results:**

Rhein significantly reduced body weight (P < 0.001), serum triglycerides (P < 0.01), and proteinuria (P < 0.001), while improving glomerular lesions. It also markedly lowered serum levels of IL-6, TNF-α, and creatinine. 16S rRNA sequencing revealed that rhein restored gut microbiota diversity (e.g., Chao1 index increased from 303.58 to 425.78) and reversed the Firmicutes/Bacteroidetes imbalance (76.86%–62.15%). Analysis of similarities (ANOSIM) further confirmed a significant difference in microbial community structure between the Rhein and Model groups (R = 0.926, p = 0.008).

**Conclusion:**

Rhein mitigates ORG progression is associated with anti-inflammatory, lipid-lowering, and microbiota-modulating mechanisms, offering a novel therapeutic strategy.

## 1 Introduction

With the global rise in obesity, chronic kidney disease (CKD) has become a growing public health concern, particularly due to the emergence of obesity-related glomerulopathy (ORG). As a major risk factor for CKD, obesity is associated with hemodynamic changes, activation of the renin-angiotensin-aldosterone system (RAAS), and dysregulation of adipokines, all of which contribute to glomerular hypertrophy and focal segmental glomerulosclerosis (FSGS), the hallmark pathological features of ORG (Fan and Jiang, 2007; Atilla and Engin, 2017; Huang et al., 2023). Despite extensive research, there remains no specific therapeutic strategy to target the underlying pathogenesis of ORG. Current treatments are primarily based on lifestyle modifications and the management of comorbid conditions such as hypertension, diabetes, and hyperlipidemia (Liu et al., 2017; Liu and Zhang, 2018; Jiang et al., 2024; Wang et al., 2025).

**FIGURE 1:**
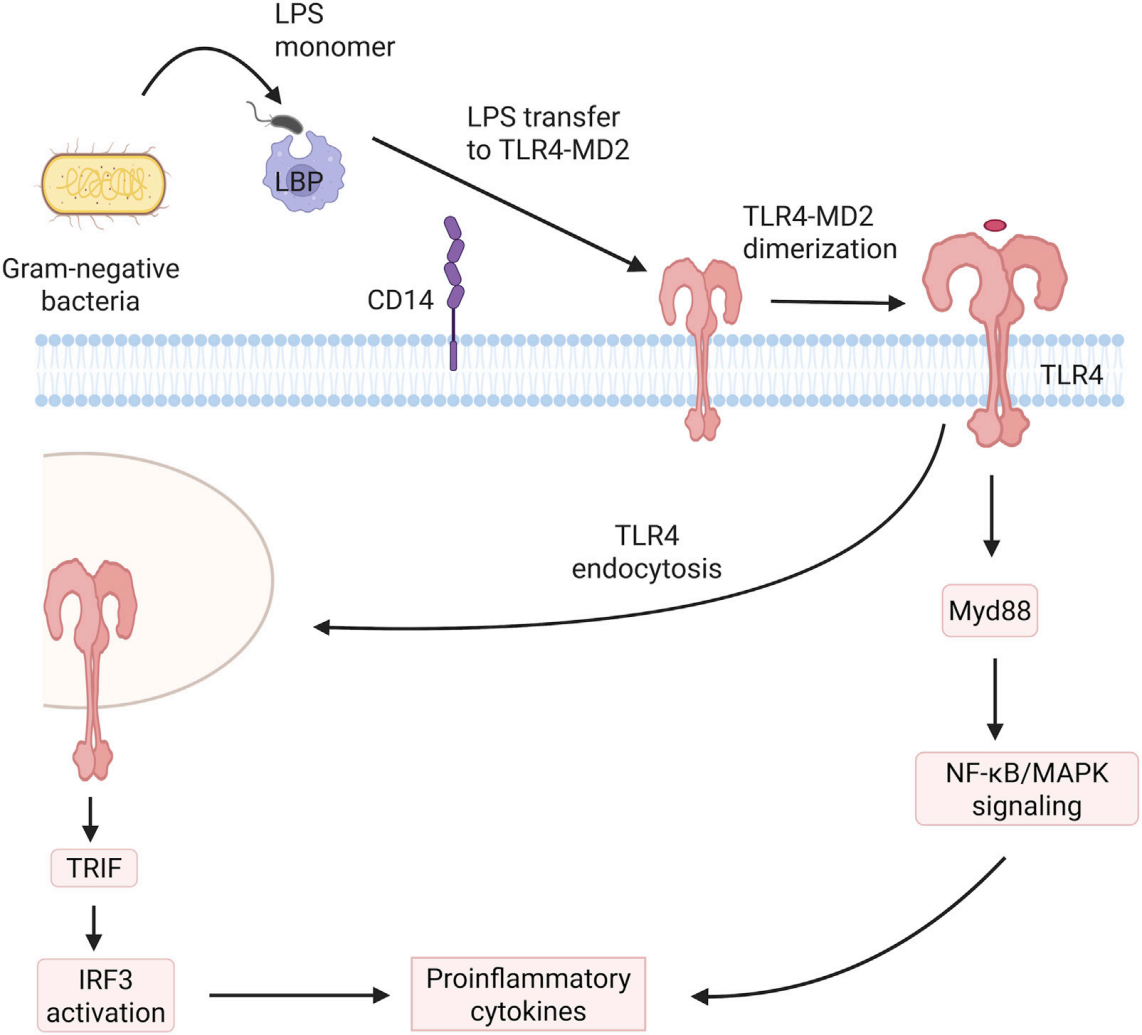
Mechanisms of gut microbiota-induced LPS and inflammatory response. LPS signaling and inflammatory activation. Lipopolysaccharide-binding protein (LBP) in serum forms specific complexes with monomeric LPS from Gram-negative bacteria, transporting the complex to CD14 receptors that exist in both soluble and membrane-associated (glycosylphosphatidylinositol-anchored) forms. The CD14-LPS interaction promotes transfer of the endotoxin to TLR4/MD-2 receptor clusters, initiating receptor dimerization. This structural rearrangement facilitates TIR domain oligomerization in TLR/IL-1R cytoplasmic regions, enabling MyD88 adaptor protein recruitment. Subsequent signaling cascades activate NF-κB and MAPK transcriptional regulators, culminating in upregulated expression of proinflammatory mediators. Concurrently, internalization of LPS-TLR4/MD-2 complexes initiate TRIF-mediated signaling that activates IRF3 transcription factor and stimulates type I interferon production.

Recent studies have highlighted the role of gut microbiota imbalance in promoting kidney injury. Disruption of intestinal flora can increase intestinal epithelial permeability, allowing bacterial metabolites such as gut-derived uremic toxins and endotoxins like lipopolysaccharide (LPS) to enter the systemic circulation (Zaky et al., 2021). This triggers low-level systemic inflammation, leading to the activation of toll-like receptors (TLR) 2 and 4, as well as the LPS receptor CD14, which in turn activates inflammatory pathways and increases the production of proinflammatory cytokines and oxidative stress (Ghanim et al., 2009; Kim et al., 2012; Figure 1).

Adipocytes secrete inflammatory mediators such as tumor necrosis factor-α (TNF-α) and interleukin-6 (IL-6), which contribute to the irreversible progression of renal fibrosis (Wang et al., 2023; Yang, 2023). Moreover, adipocytokines can directly influence glomerular cells, further exacerbating the pathogenesis of ORG (Tsuboi et al., 2013). Therefore, targeting the gut microbiota holds promising research prospects for the intervention and treatment of obesity-related kidney diseases (Hsu and Lin, 2023).

Rhei Radix et Rhizoma, first recorded in “Shen Nong’s Herbal Classic,” has been widely used in traditional medicine for its ability to delay the progression of chronic renal failure. Rhein, a principal component of Rhei Radix et Rhizoma, has demonstrated multifaceted potential in pharmacology, including anti-inflammatory, antioxidant, anticancer, antifibrotic, lipid-regulating, hypoglycemic, antibacterial, and antiviral properties (Jiao et al., 2025). Studies have demonstrated that emodin markedly enhances insulin secretion, effectively decreases blood glucose and cholesterol levels, and significantly reduces urinary albumin excretion in db/db mice. In the LPS induced acute kidney injury (AKI) model, emodin substantially suppresses the activation of the NF-κB signaling pathway and diminishes the production of inflammatory cytokines, including TNF-α and IL-6. In the APAP-induced hepatic and renal injury model, rhein mitigates oxidative stress-related renal aging and damage by inhibiting TNF-α-mediated autophagy and necroptosis, while significantly reducing oxidative stress markers such as malondialdehyde (MDA) and reactive oxygen species (ROS), and increasing antioxidant indicators such as glutathione (GSH) and superoxide dismutase (SOD) (Xiao et al., 2022; Zhu et al., 2024).

Accumulating evidence also suggests that rhein plays a role in modulating immune responses and the gut ecosystem. In rodent models of metabolic disease, rhein has been shown to alleviate systemic inflammation by inhibiting the NF-κB pathway and reducing pro-inflammatory cytokine production (Liu et al., 2023). Furthermore, emerging studies indicate that rhein can reshape the gut microbiota composition, notably by reducing the Firmicutes/Bacteroidetes ratio and enriching beneficial bacteria, which contributes to its anti-obesity and anti-inflammatory effects (Sheng et al., 2012; Chen, 2020). These properties make rhein a promising candidate for investigating the gut-kidney axis in ORG.

Despite these promising findings, the therapeutic potential of rhein in ORG, particularly through an integrated perspective that simultaneously targets the gut-kidney axis, remains largely unexplored. Most previous studies have focused on its effects in diabetic nephropathy or acute kidney injury, and a comprehensive assessment linking its metabolic, anti-inflammatory, and microbiota-modulating properties in the context of ORG is lacking.

Given the current absence of targeted therapies for ORG, this study aims to investigate the therapeutic potential of rhubarb’s active compound, rhein, in alleviating ORG using a multi-faceted approach. By elucidating the mechanisms by which rhein exerts its anti-inflammatory, antioxidant, and anti-fibrotic effects, and by specifically evaluating its impact on the gut-kidney axis, we hope to provide a novel therapeutic strategy for the treatment of this complex disease. This investigation adheres to the Four Pillars of Best Practice in Ethnopharmacology by establishing a clear ethnopharmacological rationale based on the traditional use of Rhei Radix et Rhizoma in renal diseases, focusing on a chemically defined constituent (rhein, purity >98%), employing rigorous pharmacological models to evaluate its efficacy in ORG, and providing mechanistic insights into its anti-inflammatory and microbiota-modulating activities. Based on the established pharmacological properties of rhein and the emerging role of the gut-kidney axis in ORG, we hypothesized that rhein would ameliorate renal injury in HFD-induced ORG mice by mitigating systemic inflammation and restoring gut microbiota homeostasis.

## 2 Materials and methods

### 2.1 Experimental mice and research groups

Thirty male individuals of the C57BL/6J strain mice were randomly divided into 2 groups, the control group (6 mice) and the model group (24 mice). The control group was fed a normal diet (14% fat), obtained from Shanghai Yuanye Bio-Technology Co., Ltd., and the model group was fed a high-fat diet, in which fat accounted for 60% of total calories, customized by Jiangsu Medison Biomedical Co., LTD. High-fat diet was free intake ORG modeling. After 12 weeks of feeding, the selected mice were fasted for 10 h, the 24 h urine of the mice was collected, and the mice with 24 h urinary protein ≥0.3 g/24 h were considered to be successful, the mice without successful modelling were excluded, and the mice with successful modelling were randomly divided into the model group (6 mice) with high-fat diet and the intervention group (6 mice) with rhein (purity >98%, HPLC grade), purchased from Jiangsu Suzhong Pharmaceutical Research Institute Co., LTD., the control group continued to be fed with normal diet, the model group was fed with high-fat diet, and the rhein intervention group was fed with 300 mg/kg of rhein by free intake on top of the high-fat diet, with an intervention time of 12 weeks. The dose of rhein (300 mg/kg/day) was selected based on previous studies demonstrating its efficacy in murine models of renal injury (Xiong et al., 2023) and was confirmed by our preliminary dose-ranging experiments, which indicated that this dose yielded the most pronounced improvements in metabolic and renal parameters in obese mice. This study was approved by the Ethics Committee of Fujian University of Traditional Chinese Medicine (No: FJTCM IACUC2022048).

### 2.2 Relevant inspections

#### 2.2.1 Body mass

Body mass (g) was measured at the time of grouping and every 4 weeks after rearing, and the changes in body mass of mice were recorded.

#### 2.2.2 Measurement of serum lipocalin, leptin, IL-6, TNF-α, creatinine, urea nitrogen, triglycerides

After 12 weeks of intervention, the mice were anesthetized with 1% sodium pentobarbital (100–200 mg/kg body weight), and blood samples were collected through the abdominal aorta, serum was separated, and the serum levels of adiponectin, leptin, IL-6, and TNF-α were measured using ELISA kits, while creatinine, urea nitrogen, and triglycerides were determined using standard automated biochemical assays.2.2.3 24 h protein quantification and urinary albumin.

The mice at 12 weeks of intervention were placed in washed metabolic cages, 24 h urine was collected, fasted during the retention period, no water was forbidden, 5 mL was taken after recording the urine volume and centrifuged for 10 min (centrifugation radius 10 cm) at 2,000 r/min, the sediment was removed and stored in a −20 °C cryogenic refrigerator to be measured, and the colorimetric method was applied to determine the 24 h urinary proteins, and 5 mL was taken to determine the urinary albumin.

#### 2.2.3 Histopathological examination of the kidney

Kidney tissues were routinely processed and stained with hematoxylin-eosin (HE) and proximities Schiff (PSA) for light microscopic observation of glomerular lesions. Images were captured by a digital camera of PSA-stained sections, and images were taken of the largest glomerular areas containing vascular and/or urogenital poles, which were randomly selected.

#### 2.2.4 16srRNA genome sequencing for the detection of gut microbiota in rats

Following a 12-week experimental period, fecal specimens were collected from each mice cohort and immediately transferred into sterile cryovials. Samples underwent flash-freezing using liquid nitrogen before long-term preservation at −80 °C.

For microbial analysis, fecal specimens from all mice in each group (n = 6) were analyzed. Intestinal microbiota alterations in ORG mice were investigated through 16S rRNA sequencing. The overall structural differences in microbial communities were assessed using non-metric multidimensional scaling (NMDS). Statistical Analysis of Metagenomic Profiles (STAMP) was used for intergroup bacterial community comparisons. The functional potential of the gut microbiota was predicted based on 16S rRNA sequencing data using PICRUSt2 (Phylogenetic Investigation of Communities by Reconstruction of Unobserved States). The resulting enzyme commission (EC) number abundances were normalized and visualized as a heatmap (Figure 5C). Quality assurance protocols and sequencing operations were contracted to Shanghai Meiji Biomedical Technology Co.

### 2.3 Statistical methods

Statistical analyses were performed using SPSS 25.0 software and GraphPad Prism 10 software. Measurement data were presented as mean ± standard deviation (x ± s). One-way analysis of variance (ANOVA) was used for data that met the assumptions, followed by Tukey’s *post hoc* test for multiple comparisons. For data that violated the assumptions, the non-parametric Kruskal-Wallis H test was employed, followed by the Mann-Whitney U test with Bonferroni correction for pairwise comparisons. These analyses were conducted using SPSS 25.0. GraphPad Prism 10 was used for generating the corresponding graphs. A p-value of less than 0.05 was considered statistically significant.

## 3 Results

### 3.1 The effect of rehin on renal immune inflammatory injury in ORG mice

**FIGURE 2:**
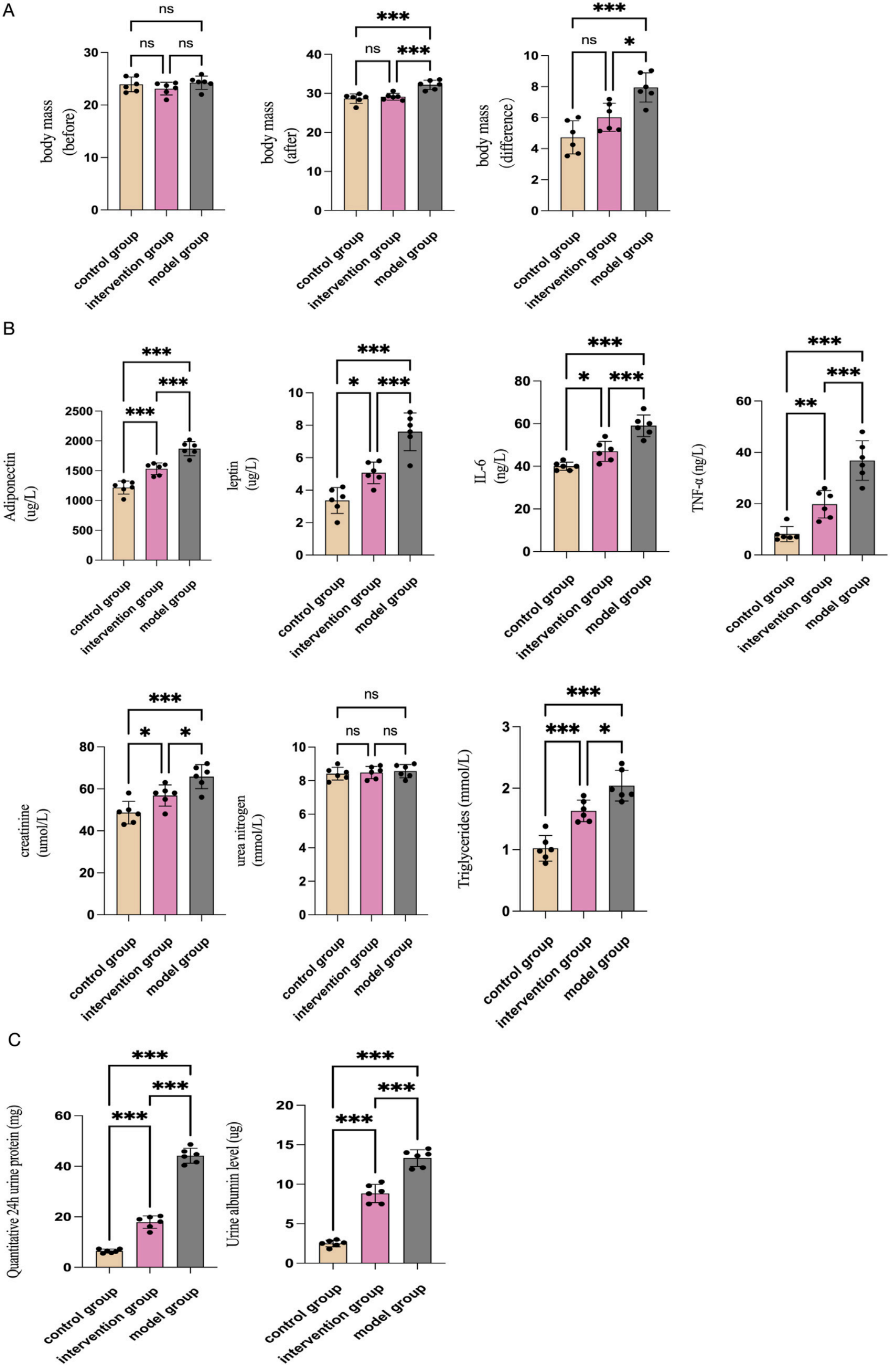
The effect of Rehin on renal immune inflammatory injury in ORG mice. **(A)** Before the intervention with rhein, the body weights of the three groups of mice were (239 ± 14) g, (243 ± 13) g, and (231 ± 12) g, respectively, with no statistically significant difference between the three groups (F = 1.237, P = 0.318). After 12 weeks of intervention, the body weights of the blank group, model group, and intervention group were (287 ± 12) g, (322 ± 12) g, and (291 ± 9) g, respectively, showing a statistically significant difference between the groups (F = 18.102, P = 0.000). The increase in body weight after intervention in the model group and intervention group was (8 ± 0.9) g and (6 ± 0.9) g, respectively, with a statistically significant difference (F = 16.531, P = 0.000). **(B)** Comparison of serum levels of adiponectin, leptin, IL-6, TNF-α, creatinine, urea nitrogen, and triglycerides in each group of mice. **(C)** Comparison of 24-h urinary protein quantification and urinary albumin content in each group of mice. N = 6. *p < 0.05, **p < 0.01, ***p < 0.001.

Firstly, we established an ORG mouse model and administered rhein via gastric lavage to evaluate its effects on ORG. Rhein significantly reduced the body weight of ORG mice (Figure 2A). Our findings indicate that rhein intervention effectively downregulated the levels of adiponectin, leptin, TNF-α, and IL-6 in the serum of ORG mice (Figure 2B). Simultaneously, we observed a decrease in serum creatinine and triglyceride expression after rhein treatment, suggesting that rhein can improve lipid metabolism and protect kidney function in ORG mice (Figure 2B). Furthermore, rhein intervention significantly reduced 24-h urinary protein excretion and urinary albumin content in mice (Figure 2C), indicating that rhein has a remarkable therapeutic effect in controlling proteinuria and protecting kidney function.

### 3.2 The effect of rhein on kidney tissue in ORG mice

**FIGURE 3:**
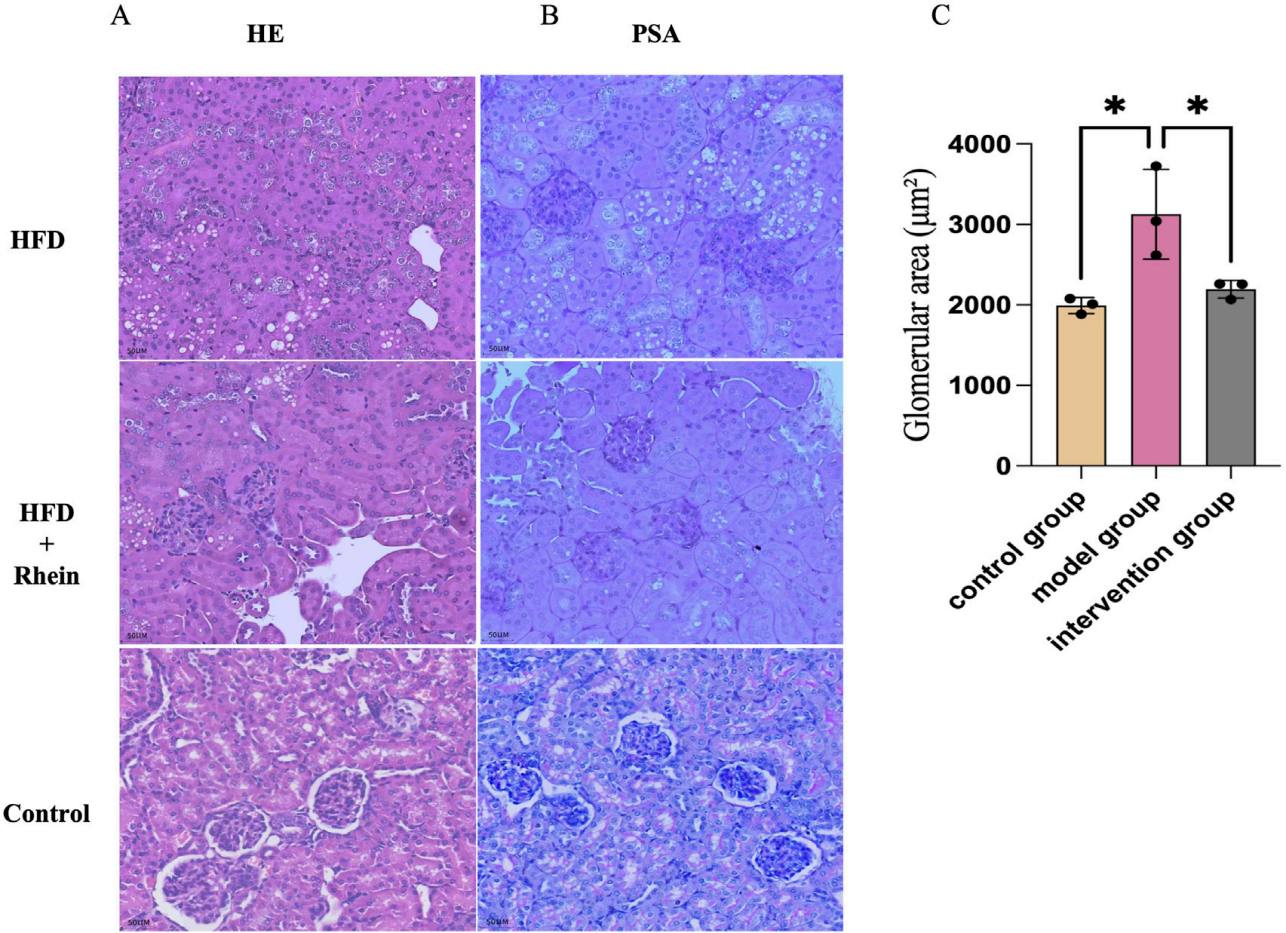
Effect of rhein on kidney tissue in ORG mice. **(A)** Renal lesions were detected by HE staining (scale bar: 50 µm, 40x). **(B)** PSA staining was used to detect renal lesions in mice (scale bar: 50 µm, 40x). **(C)** Glomerular area (μm^2^).

Histopathological analysis revealed pronounced glomerular enlargement in the model cohort, accompanied by capillary loop compression, extracellular matrix accumulation, and vacuolar alterations in renal tubular epithelium. Therapeutic intervention demonstrated measurable reductions in glomerular dimensions relative to model controls (Figure 3A). This reduction in glomerular size was quantitatively confirmed by morphometric analysis (Figure 3C). Pathological specimens from model animals exhibited substantial mesangial proliferation characterized by cellular hypertrophy, matrix deposition, and increased intraglomerular debris. Comparative evaluation showed intervention-treated subjects displayed attenuated mesangial expansion and diminished particulate accumulation within glomerular structures (Figure 3B).

### 3.3 The impact of rhein on gut microbiota diversity ORG mice

#### 3.3.1 α-diversity analysis

**FIGURE 4:**
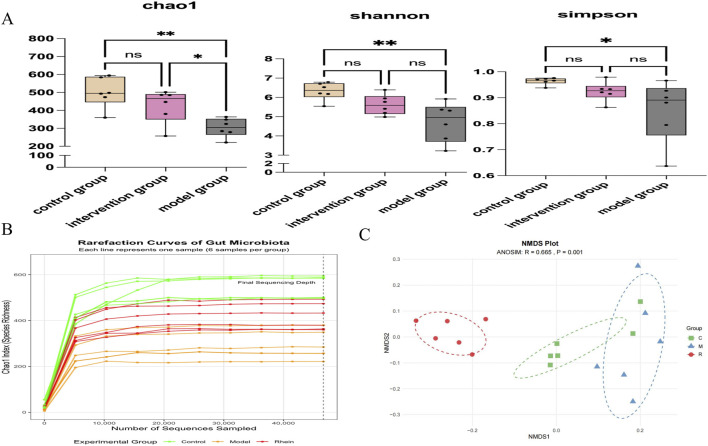
The impact of rhein on gut microbiota diversity ORG Mice. **(A)** Effect of rhein on intestinal microbiota diversity in obese rats (α-diversity analysis using Chao1, Shannon, and Simpson indices). **(B)** The Chao1 index was used as a measure of species richness for the diversity of microbial communities at different sequencing depths. **(C)** Non-metric multidimensional scaling (NMDS) plot based on Bray-Curtis distances showing the difference in overall microbial community structure among the groups. C, Control group; M, Model group; R, Rhein-intervention group. n = 6, *p < 0.05, **p < 0.01, ***p < 0.001.

As depicted in (Figure 4A), the control group exhibited a consistently high diversity index. The sample diversity of the intervention group fell between that of the control and model groups, while the model group demonstrated the lowest diversity index performance. Specifically, the Chao1 indices for the control, intervention, and model groups were (500.57 ± 85.45), (425.78 ± 93.65), and (303.58 ± 52.69), respectively. The Chao1 index was significantly lower in the model group compared to both the control group (p < 0.01) and the intervention group (p < 0.05). No significant difference was observed between the control and intervention groups (p = 0.262), suggesting that rhein intervention enhanced species richness of the gut microbiota. Additionally, there were no significant differences in the Shannon or Simpson indices between the control and intervention groups. However, significant differences were noted in the Shannon index (p = 0.004) and Simpson index (p = 0.042) between the model and control groups, with relatively small variations among all groups. These findings indicate that rhein intervention increased the species richness of the gut microbiota but failed to fully recover it to the level of the control group.

Rarefaction analysis based on the Chao1 index demonstrated that the sequencing depth of 46,668 sequences per sample was sufficient to capture most of the bacterial diversity, as all curves reached a clear plateau (Figure 4B). The Model group exhibited significantly lower species richness compared to the Control group, confirming the success of high-fat diet-induced gut microbiota dysbiosis. Importantly, Rhein intervention partially ameliorated this reduction, with the Rhein group showing intermediate species richness between the Control and Model groups.

#### 3.3.2 β-diversity analysis

Non-metric multidimensional scaling (NMDS) analysis revealed a separation in gut microbiota composition among the Control (C), Model (M), and Rhein-intervention (R) groups (Figure 4C). To statistically validate this observation, we performed an analysis of similarities (ANOSIM). The results demonstrated a significant difference in overall microbial community structure among the groups (R = 0.665, p = 0.001), indicating that the rhein intervention significantly altered the gut microbiota structure.

### 3.4 Effects of rhein on species differences and functional changes of gut microbiota in ORG mice

We next examined the changes in microbial composition at the phylum and genus levels. At the phylum level, Firmicutes, *Bacteroidota*, *Actinobacteriota*, and *Desulfobacterota* emerged as the predominant microbial phyla across all samples. Each experimental group exhibited dominance of *Firmicutes*, *Bacteroidota*, and *Actinobacteriota*, though with distinct distribution patterns. Comparative analysis revealed significant intergroup variations in phylum-level composition: *Firmicutes* showed the highest prevalence in the model group (76.86%), followed by the intervention (62.15%) and control groups (48.20%). Conversely, *Bacteroidota* demonstrated an inverse trend with maximal representation in controls (42.57%), intermediate levels in interventions (23.12%), and minimal presence in models (11.12%). *Actinobacteriota* displayed elevated proportions in the intervention group (13.79%) compared to controls (6.33%) and models (3.11%), with these variations reaching statistical significance (P < 0.05).

**FIGURE 5:**
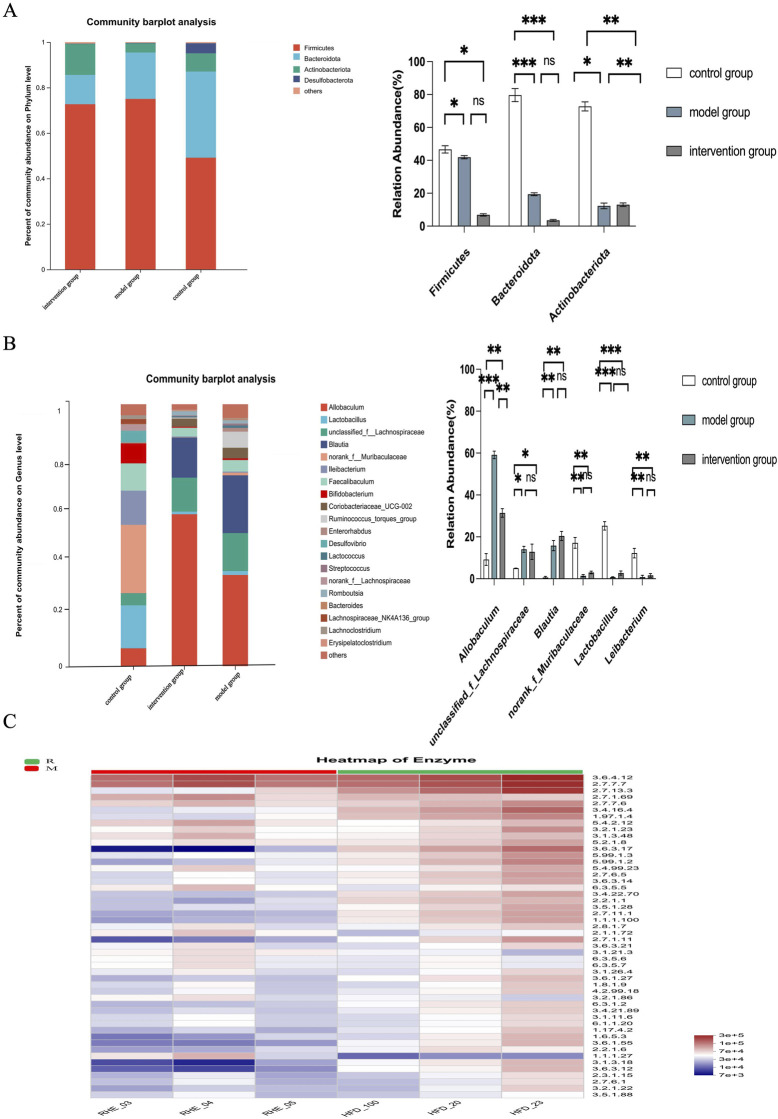
Effects of rhein on species differences and functional changes of gut microbiota in ORG mice. **(A)** Comparison of relative abundances of major differential species at phylum level. **(B)** Comparison of the relative abundances of major differential species at the genus level. **(C)** Relative abundance of enzymes in different groups. n = 6, *p < 0.05, **p < 0.01, ***p < 0.001.

Relative to the control group, both the model and intervention groups displayed increased *Firmicutes* abundance alongside reduced *Bacteroidota* levels. When compared to the model group, the intervention cohort exhibited decreased Firmicutes proportions coupled with elevated *Bacteroidota* and *Actinobacteriota* concentrations. The data delineate clear proportional relationships: Firmicutes followed a model > intervention > control hierarchy, *Bacteroidota* exhibited control > intervention > model distribution, while *Actinobacteriota* peaked in the intervention group and reached minimal levels in models (Figure 5A).

At the genus level, the ten most prevalent bacterial genera included *Allobaculum*, *Lactobacillus*, *unclassified_f_Lachnospiraceae*, *Blautia*, *norank_f_Muribaculaceae*, *Leibacterium*, *Faecalibaculum*, *Bifidobacterium*, *Coriobacteriaceae_UCG-002*, and *Ruminococcus_torques_group*. Both model and intervention groups exhibited predominant populations of *Allobaculum*, *unclassified_f_Lachnospiraceae*, and *Blautia*, while the control group was primarily characterized by *norank_f_Muribaculaceae*, *Lactobacillus*, and *Leibacterium*. Notable variations emerged in genus distribution: *Allobaculum* demonstrated the highest relative abundance in the intervention group (57.88%), followed by the model group (32.89%) and control group (7.23%). *Unclassified_f_Lachnospiraceae* showed progressive decreases from model (15.47%) to intervention (13.06%) and control groups (4.96%), whereas *Blautia* displayed maximum prevalence in the model group (21.98%) compared to intervention (17.56%) and control groups (0.23%).

Conversely, *norank_f_Muribaculaceae* dominated the control group (18.98%) with minimal presence in model (2.56%) and intervention groups (1.83%). *Lactobacillus* exhibited a similar pattern with control group dominance (23.86%) versus model (2.04%) and intervention groups (0.88%). *Leibacterium* prevalence followed this trend with control group superiority (13.84%) over model (0.98%) and intervention groups (0.45%). Relative to controls, both model and intervention groups demonstrated substantial microbial shifts including elevated *Allobaculum*, *unclassified_f_Lachnospiraceae*, and *Blautia* alongside reduced *norank_f_Muribaculaceae*, *Lactobacillus*, and *Leibacterium*. The intervention group specifically showed *Allobaculum* enrichment but diminished *unclassified_f_Lachnospiraceae* and *Blautia* compared to model group. These findings highlight *Allobaculum* as the predominant genus in intervention cohorts, *Blautia* in model groups, and *norank_f_Muribaculaceae* in controls. Comparative analysis revealed consistent microbial distribution patterns between model/intervention groups that differed significantly from controls (Figure 5B).

Functional heatmap analysis revealed distinct changes in enzyme activity across different groups. A significant difference in the metabolic profiles of the intestinal flora was observed between the model group and the intervention group, as indicated by the altered expression of key metabolic enzymes. These findings are further supported by the data presented in (Figure 5C). The altered enzyme activities predicted by PICRUSt2 suggest shifts in the metabolic capabilities of the gut microbiota. Specifically, changes were noted in EC numbers related to carbohydrate and amino acid metabolism, hinting at potential alterations in short-chain fatty acid (SCFA) biosynthesis and other metabolic pathways crucial for host-microbe interaction. Changes in enzymes involved in short-chain fatty acid (SCFA) metabolism or amino acid fermentation could influence the production of key metabolites that modulate host inflammation and energy homeostasis, which are central to ORG pathogenesis. Rhein’s intervention appears to normalize these aberrant metabolic pathways, providing a preliminary, hypothesis-generating link between the observed microbiota changes and the improved host phenotype.

## 4 Discussion

In this study, we demonstrated that the natural compound rhein effectively alleviates the progression of obesity-related glomerulopathy (ORG) in a mouse model. Our findings provide evidence that its therapeutic actions are associated with the concurrent amelioration of systemic inflammation, improvement of lipid metabolism, and restoration of gut microbiota homeostasis. Specifically, rhein treatment significantly reduced body weight gain, serum levels of triglycerides, pro-inflammatory cytokines (TNF-α, IL-6), and proteinuria, while also attenuating glomerular injury.

Our findings show that rhein significantly improves inflammatory markers and lipid profiles in ORG mice. Rhein affects lipid profiles in mice, significantly lowering serum triglyceride levels and reducing body weight, thereby improving carbohydrate and fatty acid metabolism. The decrease in body mass as well as the attenuation of inflammatory factor release induced by adipocytes may be associated with the downregulation of specific transcription agents such as PPARγ and C/EBPα, and their upstream regulator C/EBPβ, thereby inhibiting the process of adipogenesis (Sheng et al., 2012; Dong et al., 2016). Previous studies have consistently demonstrated that rhein exerts anti-obesity effects by suppressing the expression of these key adipogenic transcription factors and improving lipid metabolism (Sheng et al., 2012; Dong et al., 2016; Zhou et al., 2024). This point will be further confirmed in our subsequent research. Furthermore, in the treatment of ORG mice with rhein intervention, the reduction of urinary protein is more pronounced compared to the model group. Simultaneously, pathological manifestations indicate that rhein has a certain degree of inhibitory effect on glomerular mesangial matrix proliferation. Therefore, rhein can be considered as a therapeutic agent that improves systemic inflammation, controls proteinuria, and exhibits significant efficacy in the treatment of ORG.

Emerging research indicates that elevated intestinal permeability in obese rodent models facilitates significant translocation of lipopolysaccharide (LPS) into systemic circulation. This endotoxin is known to trigger the TLR-4-mediated signaling pathway, subsequently stimulating macrophages to secrete pro-inflammatory mediators including TNF-α and IL-6 (Kim et al., 2012). Our observed reduction in these cytokines is consistent with the potential suppression of this pathway, although direct molecular evidence from our study is lacking. These molecular events foster a chronic inflammatory milieu that disrupts metabolic homeostasis, potentially exacerbating obesity-related complications and insulin signaling dysfunction (Tian et al., 2024). The gut microbial ecosystem demonstrates intricate connections with adiposity pathogenesis, particularly through dysbiosis patterns characterized by reduced microbial diversity and altered community structures. Experimental animal studies frequently report microbial population shifts quantified through elevated *Firmicutes/Bacteroidetes* ratios (F/B) (Vallianou et al., 2019; Avgoustou E et al., 2025). Current investigations emphasize therapeutic strategies targeting gut microbiota modulation to mitigate ORG progression. Aligned with the “gut-kidney axis” hypothesis, our research evaluates Rhein’s potential as a microbial modulator for delaying renal pathology in ORG models.

Experimental findings demonstrate that ORG mice exhibit diminished diversity in their gut microbial communities, while rhein supplementation enhances bacterial abundance within this model. Beta-diversity assessments reveal that rhein administration induces substantial microbial community restructuring, with clear compositional variations observed between experimental cohorts. Existing literature documents that obesity-associated dysbiosis typically features elevated populations of pro-adipogenic and inflammatory microbial taxa alongside diminished anti-inflammatory species (Wei et al., 2018; Cai et al., 2023). Notably, microbial profiles show elevated Firmicutes levels coupled with reduced Bacteroidetes representation. This altered *Firmicutes/Bacteroidetes* ratio enhances dietary energy harvest efficiency and facilitates subcutaneous adipose tissue deposition (Turnbaugh et al., 2006; Meijnikman et al., 2020; U et al., 2023). Among Bacteroidetes members, Prevotella strains demonstrate intestinal barrier reinforcement capabilities through succinate-mediated enhancement of tricarboxylic acid cycle activity, thereby supporting glycemic regulation. Such functional attributes position these microbes as promising candidates for advanced probiotic development (Le Chatelier et al., 2013; Huang et al., 2021; Wang et al., 2022). Analysis of 292 participants demonstrated that enteric microbial gene content and species diversity effectively stratify populations. A “Low Gene Count” subgroup (23% of participants) contained disproportionate numbers of obese individuals, displaying marked systemic inflammation, severe adiposity, impaired insulin sensitivity, and lipid metabolism abnormalities (Le Chatelier et al., 2013).

While obesity remains the primary factor in ORG pathogenesis, this investigation revealed distinct microbial shifts between experimental groups. The ORG model cohort demonstrated a notable rise in *Firmicutes* abundance accompanied by diminished Bacteroidetes populations. Contrastingly, rhein-administered subjects displayed reversed trends with suppressed Firmicutes levels and enhanced *Bacteroidetes* colonization. Taxonomic analysis at genus resolution exposed differential distribution patterns-*Blautia*, predominated in model specimens, whereas therapeutic intervention stimulated *Bacteroides* proliferation alongside *Blautia* attenuation (Wei et al., 2018; Cai et al., 2023). As gram-negative intracellular pathogens, *Blautia* species employ specialized LPS configurations to evade immune surveillance, establishing protected niches while paradoxically inducing chronic inflammatory cascades. Sustained immune activation from such stealth pathogens drives progressive tissue damage through cytokine-mediated pathways. Crucially, rhein therapy in ORG murine models effectively suppressed *Blautia* colonization while amplifying beneficial *Bacteroides* populations associated with mucosal barrier fortification. These microbial modulations suggest rhubarb-derived compounds may exert therapeutic effects through dual mechanisms: fostering probiotic expansion while constraining pathogenic overgrowth. Experimental evidence supports the hypothesis that rhein administration rectifies diet-induced microbial dysbiosis in ORG models, potentially restoring intestinal barrier integrity and mitigating inflammatory progression, thereby decelerating disease advancement.

Although the present study did not directly measure microbial metabolites such as short-chain fatty acids (SCFAs), the observed structural changes in the gut microbiota align well with functional alterations reported in the literature. For instance, an increased *Firmicutes/Bacteroidetes* (F/B) ratio is often associated with enhanced energy harvest and an obese phenotype (Turnbaugh et al., 2006). The significantly elevated F/B ratio in the model group (Model > Intervention > Control) conforms to this pattern. The reduction in the F/B ratio following Rhein intervention suggests that it may ameliorate obesity by modulating the microbiota structure to reduce energy absorption. This represents a plausible, though correlative, mechanism.

More importantly, bacteria belonging to the phylum *Bacteroidetes* are primary producers of beneficial SCFAs, such as acetate and propionate (Evenepoel et al., 2017; Rios-Covian et al., 2020). SCFAs serve not only as energy substrates but also exert multiple benefits, including anti-inflammatory effects, maintenance of gut barrier integrity, and improvement of insulin sensitivity, through mechanisms such as activation of G-protein-coupled receptors (GPR43, GPR109A) and inhibition of histone deacetylases (HDACs) (Parada Venegas et al., 2019). Therefore, the relative increase in Bacteroidetes abundance after Rhein intervention may imply increase SCFA production, which is crucial for alleviating systemic low-grade inflammation and renal injury in ORG.

Conversely, genera enriched in the model group, such as *Blautia*, have been associated with inflammatory states in inflammatory bowel disease and metabolic disorders (Liu et al., 2021). Some taxa may also be involved in producing precursors of uremic toxins, such as indoxyl sulfate (IS) and p-cresyl sulfate (PCS), which exacerbate renal damage and fibrosis (Evenepoel et al., 2017). The reduction in *Blautia* abundance following Rhein treatment may indirectly decrease the production of these harmful metabolites, thereby reducing the toxin load on the kidneys. In summary, Rhein likely protects against ORG via the ‘gut-kidney axis’ by remodeling the gut microbiota ecosystem, promoting the production of beneficial metabolites (e.g., SCFAs), and simultaneously suppressing harmful metabolic pathways.

Furthermore, the functional potential of the gut microbiota, predicted by PICRUSt2 analysis, revealed distinct patterns of enzyme commission (EC) abundance between the model and intervention groups (Figure 5C). The altered profile in the rhein-treated group suggests a shift in the collective metabolic capabilities of the microbial community. Specifically, changes were noted in EC numbers related to carbohydrate and amino acid metabolism, hinting at potential alterations in short-chain fatty acid (SCFA) biosynthesis and other metabolic pathways crucial for host-microbe interaction. While these predictions require direct metabolic validation, they provide a preliminary, hypothesis-generating link between the observed structural changes in the microbiota and the improved host phenotype.

In summary, the mechanism of action of rhein in obesity-related nephropathy, as revealed by this study, involves multiple levels: it inhibits systemic inflammatory responses, reduces proteinuria, promotes lipid metabolism, and modulates the intestinal flora structure. The remodeling of the gut microbiota ecosystem provides a compelling and associative mechanism within the ‘gut-kidney axis’ for Rhein’s protective effects against ORG. However, this study primarily establishes a correlation between microbiota changes and improved phenotype. Further exploration using metabolomics, targeted molecular studies on signaling pathways, and interventional experiments like fecal microbiota transplantation is needed to definitively identify the functional metabolites and specific targets of rhein in preventing and treating ORG, which will provide deeper insights into its clinical application.

## 5 Limitations and future perspectives

This study has some limitations. Firstly, the use of a single dose of rhein, although justified, precludes a full dose-response analysis. Secondly, food intake was not systematically monitored, which is a recognized limitation in dietary intervention studies. Thirdly, the mechanistic insights are primarily based on phenotypic and correlative data. While our findings are consistent with the involvement of inflammatory pathways such as TLR4/NF-κB, direct molecular evidence for their inhibition in the kidney was not obtained and remains an important goal for future research. Future studies should include molecular analyses of key signaling pathways and employing pair-feeding designs will be valuable to conclusively dissect the contribution of caloric intake versus specific drug effects. Nonetheless, the significant reversal of ORG phenotypes in rhein-treated mice while maintained on a high-fat diet underscores its therapeutic potential.

## Data Availability

The data are available in the OMIX repository, accession number OMIX013033. Available at: https://ngdc.cncb.ac.cn/omix/release/OMIX013033.

## Supplementary material

SUPPLEMENTARY TABLE S1 Biochemical parameters of the experimental mouse groups. Data are presented as mean ± SD. IL-6, interleukin-6; TNF-α, tumor necrosis factor-α. ^a^P < 0.05, ^c^P < 0.01 vs. model group; ^b^P < 0.05, ^d^P < 0.01 vs. control group.
