# Supplementary material for: Rhein ameliorates inflammation, gut dysbiosis, and renal injury in obesity-related glomerulopathy mice
Source: Front Pharmacol. 2025 Dec 4;16:1654062. doi: 10.3389/fphar.2025.1654062 (PMC12712377; doi:10.3389/fphar.2025.1654062)
Supplement: Supplementary file 1 [file Table1.docx]

| Group | Adiponectin  （ug/L） | Leptin  （ug/L） | IL-6  （ng/L） | TNF-α  （ng/L） | Creatinine  （umol/L） | Urea nitrogen  (mmol/L) | Triglycerides (mmol/L) | 24h urinary protein quantification（mg） | Urinary  microalbumin  (ug) |
| --- | --- | --- | --- | --- | --- | --- | --- | --- | --- |
| Control | 1218±108 | 3.37±0.79 | 40±1.90 | 8.15±2.94 | 48.67±5.35 | 8.42±0.38 | 1.02±0.21 | 6.45±0.76 | 2.50±0.42 |
| Model | 1870±119^d^ | 7.60±1.16^d^ | 59±5.06^d^ | 36.83±7.70^d^ | 65.83±5.74^d^ | 8.57±0.39 | 2.04±0.25^d^ | 44.18±2.96 | 13.32±1.06 |
| Intervention | 1540±292^cd^ | 5.07±0.66^cd^ | 47±4.73^bc^ | 19.77±5.34^cd^ | 56.83±5.74^ab^ | 8.48±0.37 | 1.63±0.17^cd^ | 17.88±2.48 | 8.83±1.16 |
| F | 53.775 | 33.792 | 32.209 | 38.805 | 15.255 | 0.236 | 34.680 | 435.619 | 200.440 |
| P | 0.000 | 0.000 | 0.000 | 0.000 | 0.000 | 0.792 | 0.000 | 0.000 | 0.000 |

Table S1 All biochemical data (mean ± SD) for each group.

Note: IL-6 is interleukin 6; TNF-α is tumor necrosis factor α; compared with the model group, ^a^P < 0.05, ^c^P < 0.01; compared with the control group, ^b^P < 0.05, ^d^P < 0.01.
